# Psychrophilic *pseudomonas* in antarctic freshwater lake at stornes peninsula, larsemann hills over east Antarctica

**DOI:** 10.1186/s40064-015-1354-3

**Published:** 2015-10-07

**Authors:** Abhishek Chauhan, Pawan K. Bharti, Pankaj Goyal, Ajit Varma, Tanu Jindal

**Affiliations:** Amity Institute of Environmental Toxicology, Safety and Management, Amity University, Sector-125, Noida, Uttar Pradesh India; Antarctica Laboratory, R & D Division, Shriram Institute for Industrial Research, 19, University Road, Delhi, 110007 India; Amity Institute of Microbial Technology, Amity University, Sector-125, Noida, Uttar Pradesh India

**Keywords:** Psychrophilic *Pseudomonas*, Antarctic freshwater lake, East Antarctica, Stornes peninsula

## Abstract

The Larsemann Hills is an ice-free area of approximately 50 km^2^, located halfway between the Vestfold Hills and the Amery Ice Shelf on the south-eastern coast of Prydz Bay, Princess Elizabeth Land, East Antarctica (69º30′S, 76º19′58″E). The ice-free area consists of two major peninsulas (Stornes and Broknes), four minor peninsulas, and approximately 130 islands. The Larsemann Hills area contains more than 150 lakes at different Islands and Peninsulas. Nine lake water samples were collected in a gamma sterilized bottles and were kept in an ice pack to prevent any changes in the microbial flora of the samples during the transportation. The water samples were transported to the lab in vertical position maintaining the temperature 1–4 °C with ice pack enveloped conditions. Samples were studied for Psychrophilic bacterial count, *Pseudomonas* spp., *Staphylococcus aureus*, *Salmonella* and Total MPN Coliform per 100 ml. Psychrophillic counts were found in the range of 12 cfu to 1.6 × 10^2^ cfu in all the samples. MPN Coliform per 100 ml was found to be absent in all the samples. No growth and characteristics colonies observed when tested for *Salmonella* and *S.aureus.Pseudomonas* sp. was found in ST-2 lake water sample as characteristics colonies (Optimum Growth) were observed on selective media at 22 and 25 °C. Further several biochemical tests were also performed to confirm the presence of this Potential Psychrophilic *Pseudomonas* sp. for its further application in Science and Technology.

**Electronic supplementary material:**

The online version of this article (doi:10.1186/s40064-015-1354-3) contains supplementary material, which is available to authorized users.

## Background

Antarctica has for a long time been viewed as a pristine and isolated environment mostly untouched by human activity. Environmental conditions are generally unfavorable in terrestrial Antarctic environments, with low thermal capacity of the substratum, frequent freeze–thaw and wet-dry cycles, low and transient precipitation, reduced humidity, rapid drainage and limited organic nutrients. Despite the extreme conditions, microorganisms including bacteria, archaea, micro fungi and microalgae, are the dominant life form in the Antarctic ecosystems representing relatively simplified system sensitive to perturbations (Niederberger et al. 2008; Gesheva 2009; Gesheva and Negoita 2012). Psychrophilic and psychrotolerant microorganisms have developed various structural and functional adaptations allowing them to survive in harsh environments. In view of the severe environmental conditions prevailing in Antarctica, it has been argued that the production of extracellular compounds would be a particular advantage in reducing inter-species competition. Thus, it was suggested that Polar Regions may be viewed as a vast untapped reservoir of microorganisms with manifold potential application (Manan et al. 2014). Scientists especially microbiologists throughout the world focusing on cold adopted microorganisms known as *Psychrophiles* which optimally grow at 15 °C. Cold adopted microorganisms are also reported for the effective bioremediation of oil polluted alpine soils (Margesin 2000). Reddy et al. (2003) and Mountfort et al. (1997) have done the extensive work for the microbial life in the polar environment and have been discovered and described new taxa of Psychrophiles. Several strains of bacteria have been isolated from extreme environments of Antarctica. Gram negative spore-forming, non-spore forming rods, Gram positive cocci were isolated from the snow, ice, and frozen algae of Antarctica (McClean 1918). Temperature-sensitive molecules and enzymes of psychrophiles have applications in pharmaceuticals. The cryoprotectors synthesized by psychrophilic and psychrotolerant microorganisms are used in agriculture, cosmetics, and medicine. Lakes of Antarctica represent a unique ecosystem and not as much considered then lowland lakes mainly because of their remoteness and the short summer open-water period (Bhat et al. 2011).Although they are protected from direct human impact but from last two decades they are largely affected by airborne contaminants, such as acids and nutrients (Rogora et al. 2006), organic pollutants and heavy metals (Carrera et al. 2002). Bharti and Gajananda (2013) reported that even less impact on soft water high altitude lakes might affect significantly the physical and chemical properties to bring changes in species symphony and plenty of the biota and to origin addition of trace substances in higher trophic microorganisms.

The revival of cultivable microorganisms from lakes of Antarctica is not easy; therefore by using rapid and internationally approved method it is possible to understand the diversity, survival, and activity of microorganisms in Antarctic zone. Darling and Siple (1941) isolated 178 strains from the snow, ice, soil and debris of Antarctica. Cultivation and characterization of microorganisms in Antarctic Lakes have also been studied by Zhang et al. 2008. Molecular approach was also adopted for further identification of Antarctic microorganisms. Reddy et al. (2004) evaluated several samples of cyanobacterial mat collected from various water bodies in Antarctica and isolated thirty-one bacteria that belonged to the genus *Pseudomonas*. All the isolates were found psychrophilic and out of them three novel species of the genus *Pseudomonas* as *Pseudomonas antarctica* sp. nov., *Pseudomonas meridiana* sp. nov. and *Pseudomonas proteolytica* sp. nov., identified based on Phenotypic and chemotaxonomic characteristics. Richard et al. (2009)studied the microbiology of polar environment and also reported the cultural conditions, nutritional adaptability of Psychrophiles and other novel microorganisms. Present study was designed to identify the microbiological contamination in the lake samples collected during 30th Indian Scientific Expedition to Antarctica. Microbiological Parameters as per the Indian standard of drinking were selected and Emphasis was given to isolate the psychrophilic microorganisms keeping in view the environmental condition of Antarctica.

## Methods

### Sampling site

**Fig. 1:**
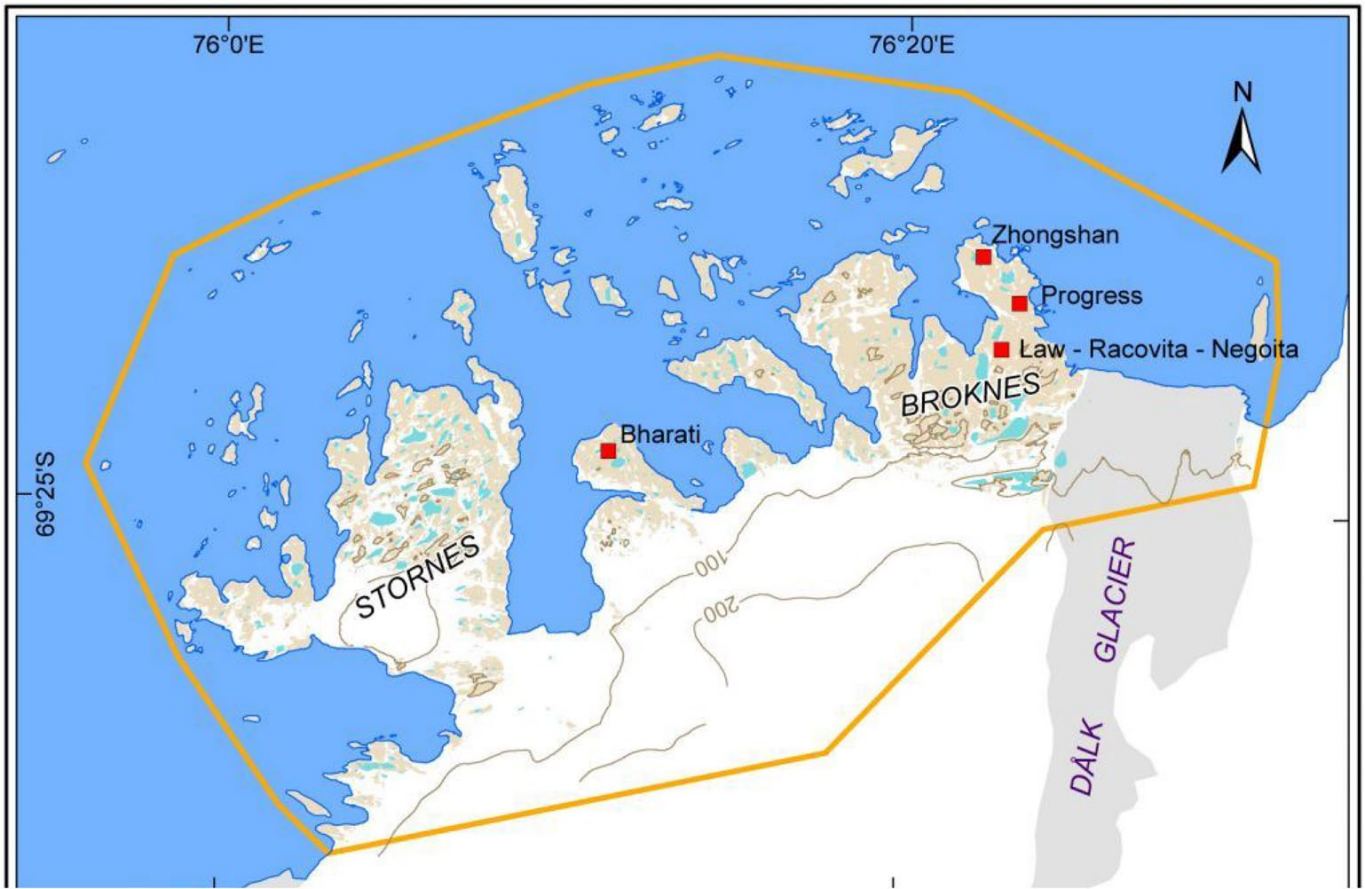
Location map of Stornes peninsula in Larsemann Hills, east Antarctica

Stornes Peninsula of Larsemann Hills in east Antarctica was selected as a sampling site for the present study (Fig. 1). Water samples were collected from two selected lakes of Stornes Peninsula during 30th Indian Scientific Expedition to Antarctica (ISEA).

### Method of collection of water samples

Gamma irradiated, clean and sterilized bottles (200 ml capacity) were used for the collection of lake water sample from Antarctica. For dechlorination sodium thiosulphate was added to the clean, dry sampling bottles before gamma sterilization in an amount to provide an approximate concentration of 100 mg/lit in the sample (Chauhan et al. 2015). Aseptic conditions were maintained during the collection of samples. The samples were kept in an ice pack to prevent any changes in the microbial flora of the samples during the transportation. The water samples were transported to the lab in vertical position maintaining the temperature 1–4 °C with ice pack enveloped conditions.

### Media, chemical and reagents

All the media were procured form Hi-Media Laboratory, Mumbai, India. Procured dehydrated media were used as per the instructions written on the box and growth promotion test of each media carried out before evaluation of samples. Sodium Chloride, Sodium thiosulphate and other chemicals were of analytical grade.Gram-Stain kit and other reagent procured from Difco Laboratories.

### Method for the determination of psychrophilic bacterial count

Serial Dilution Pour plate Method was used for the determination of *Psychrophilic* Bacterial Count. After proper mixing, collected sample was serially diluted up to 10^7^ dilutions. Diluted sample were further inoculated (1 ml) to each Petri dishes in duplicate. About 15 –20 ml melted media Plate count agar was used for *Psychrophilic* Bacterial Count as per the guidelines of Indian Standard (IS: 5402-2002, Reaff: 2007). After solidification plates were incubated in an inverted position at 22 °C for 24 h. After incubation the plates were observed for the bacterial colonies with the help of Quebec Colony Counter and then calculated in terms of cfu per ml.

### Enumeration of coliform bacteria (MPN Coliform/100 ml)

The most probable number (MPN) technique as per IS1622:1981 was implemented for the enumeration of total coliform. The test procedure included three phases namely presumptive, confirmative and completed phase.

### Presumptive test

For each water sample; 5 tubes of each 10, 1, and 0.1 ml were used. 10 ml sample was inoculated in double strength Mc Conkey broth media and rest 1 and 0.1 ml was inoculated in single strength Mc Conkey broth media. All the inoculated tubes were incubated at 37 °C for 24–48 h. The test for Coliform is discontinued for further as no growth in all MacConkey broth tubes observed.

### Method of detection of *Salmonella* sp

For the detection of *Salmonella.* 250 ml water sample was passed through 0.45 micron filter and the filter paper was inoculated in buffer peptone water and then incubated at 37 °C for 24 h. 0.1 ml of above enriched sample was inoculated in 10 ml of Rappaport–Vassiliadis (RV) medium and then incubated at 42 °C for 24 h. Subcultured on the plates of two selective media such as brilliant green agar and bismuth sulphide agar. Plates were observed for characteristic colonies such as pink colonies on brilliant green agar and black metallic sheen colonies with H_2_S on bismuth sulphide agar plates. Further confirmation through Biochemical and Serological tests was discontinued as no characteristics colonies observed on the plates of selective media (IS: 5887(Part-3) 1999 Reaff.2005).

### Method of detection of *Staphylococcus aureus*

For the detection of *Staphylococcus aureus* 250 ml water sample was passed through 0.45 micron filter and the filter paper was inoculated in cooked medium and then incubated at 37 °C for 24 h. Subcultured on the Mannitol salt agar and Baird parker agar plates. Plates were observed for characteristic colonies such as yellow colonies on Mannitol salt agar plates and black colonies on Baird parker agar plates. Further confirmation through Biochemical and Serological tests was discontinued as no characteristics colonies observed on the plates of selective media IS: 5887(Partt-2) 1976 Reaff.2005.

### Method of detection of *Pseudomonas aeruginosa*

For the detection of *Pseudomonas sp.* 250 ml water sample was passed through 0.45 micron filter and the filter paper was inoculated in cetrimide broth and then incubated at 4, 15, 22, 25, 37 °C for 48 h in individual tubes. Subcultured on the plates of cetrimide agar. Plates were observed for characteristic green colonies, optimum growth were observed at 22 and 25 °C. Further confirmation was done by Gram’s staining and Biochemical test as per the guidelines of IS: 13428:^2005^ (Additional file 1: Table S1).

## Results and discussion

**Fig. 2:**
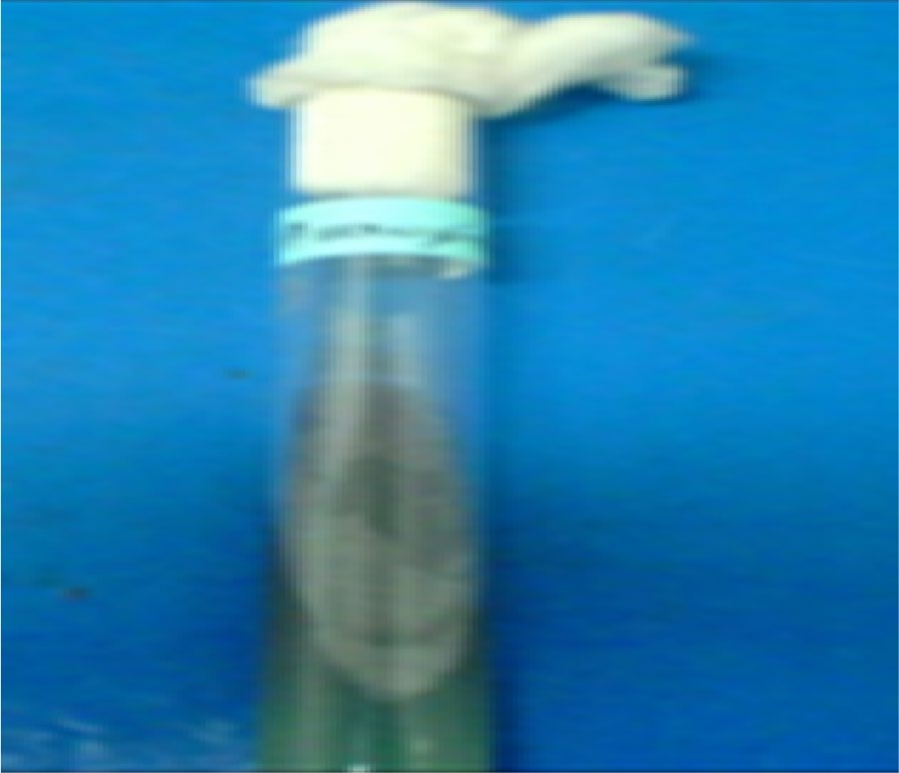
Growth of isolate on nutrient agar slant

**Fig. 3:**
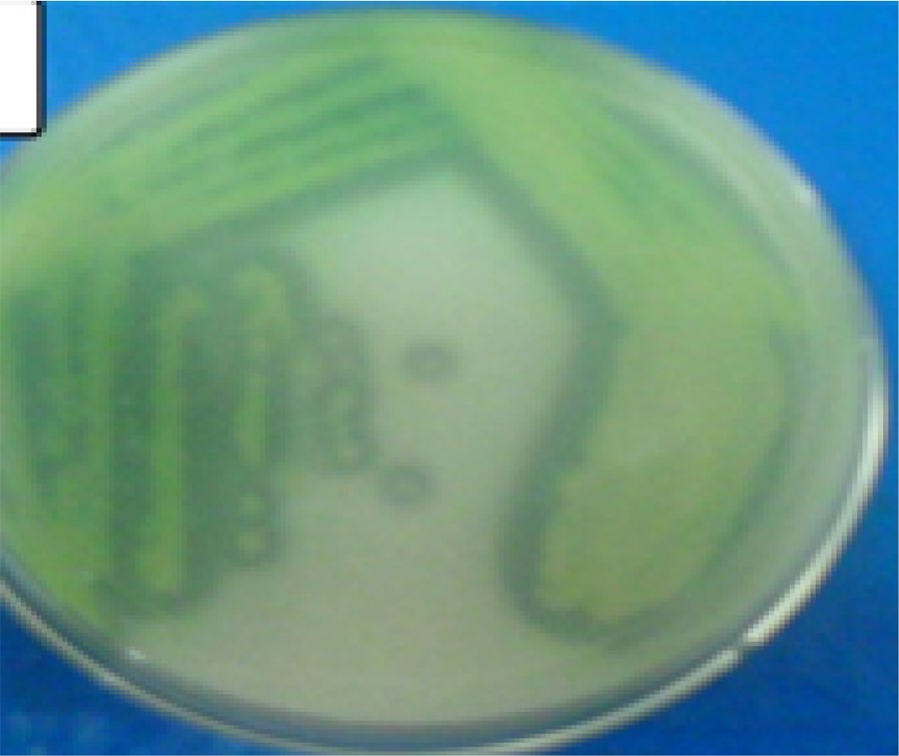
Characteristics colonies on Cetrimide agar (a selective agar media)

**Fig. 4:**
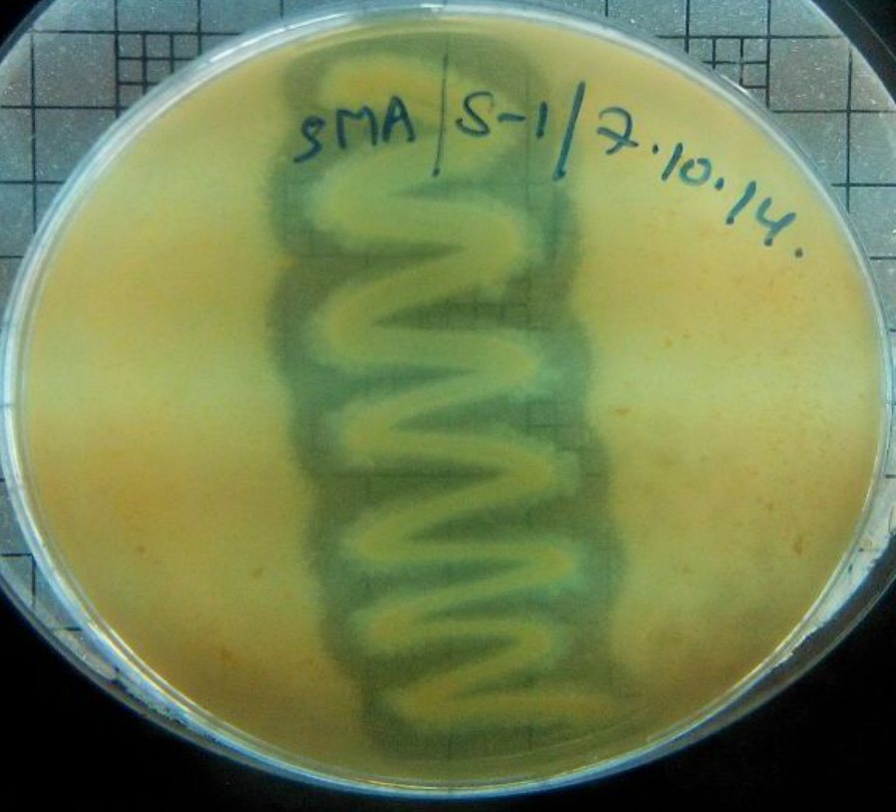
Casein hydrolysis on skimmed milk agar

**Fig. 5:**
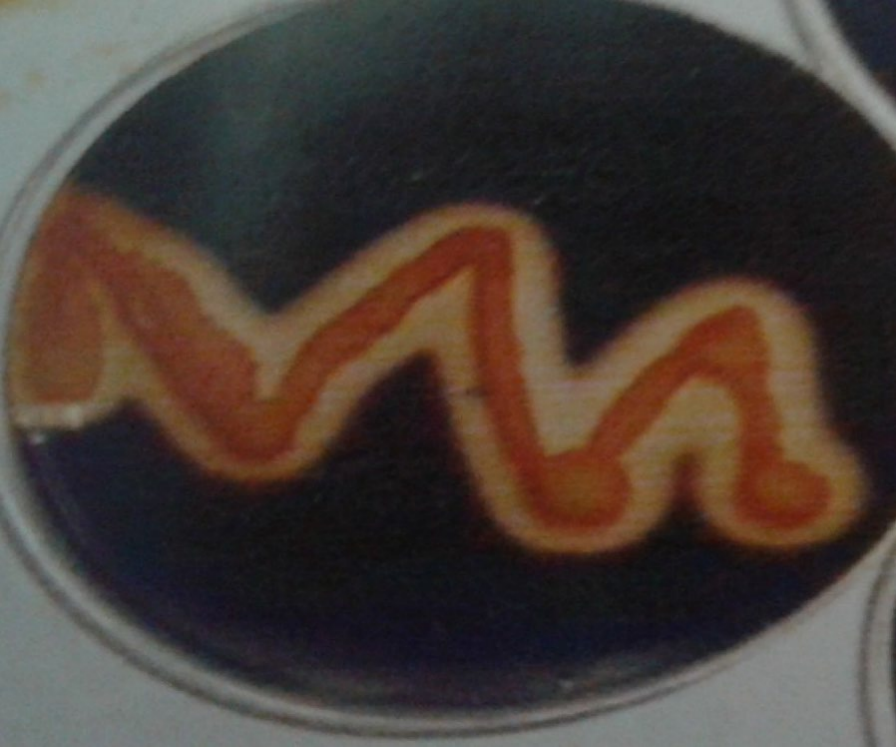
Starch hydrolysis on starch agar

**Fig. 6:**
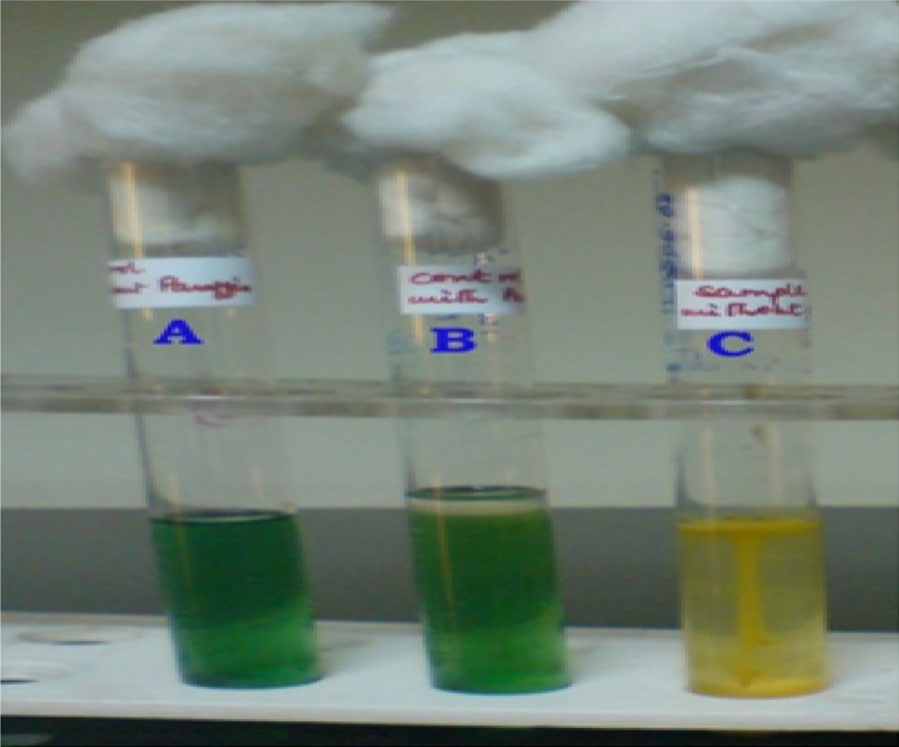
Hugh and Leifson test: *A* Media control tube. *B* Tube having the liquid paraffin (no growth). *C* Tube shows the yellow growth (Oxidative)

**Fig. 7:**
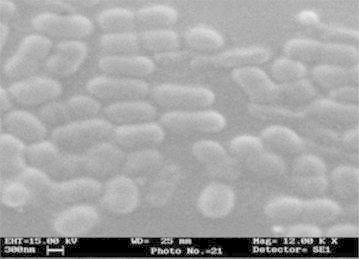
Scanning electron micrograph (SEM) image of Isolated *Pseudomonas* sp.

The present research study was carried out to know the microbiological contamination in Antarctic freshwater lake at stornes peninsula, larsemann hills over east Antarctica. Data of collected sample is summarized in Additional file 1: Table S1 and Additional file 2: Table S2. Psychrophillic counts were found in the range of 12 cfu to 1.6 × 10^2^ cfu in all the samples. MPN Coliform per 100 ml was found to be absent as No Growth in presumptive test was observed, hence sample discontinued for further studies. No any growth and characteristics colonies observed when tested for *Salmonella* and *S. aureus.Pseudomonas* sp. was found in ST-2 lake water sample as characteristics colonies (optimum growth) were observed on selective media at 22 and 25 °C. Isolated Pseudomonas was further study for its morphological identification. The isolate was identified Gram-negative rod shaped in Gram-staining slide under Laboratory Microscope. Several biochemical tests were carried out for further identification of isolated strain. The observation of all biochemical test of *Pseudomonas* isolate is given in Additional file 1: Table S1; Figs. 1, 2, 3, 4, 5, 6 and 7. A positive strain of *Pseudomonas aeruginosa* MTCC 1688 was also used as a positive control to ensure the laboratory condition during biochemical identification isolated strain. Our study is supported by the research work done by Zhang et al. (2008). They had reported the homology analysis of isolates from Antarctica and identified several close representative group of microorganisms. They also studied the colony morphological characteristics of isolated microorganisms and found that mostly isolates were growing at 4 °C which also an indicator of presence of Pstchrophilic microorganisms in Antarctica.

## Conclusion

With increasing amounts of research taking place it is becoming clear that Antarctica not only profoundly influences us, but that we are globally linked and are having an influence on it too through microbial pollution produced locally and from all over the globe. Most human activity and its associated pollution in Antarctica is highly localized, and waste disposal in the early years was in the form of snow pits, waste dumps, open pit burning, and release of untreated sewage into the oceans. This was the mindset in those times, but has now changed to a more environmentally aware regime. Antarctica is now facing a more serious threat from microbial pollution which began at the time of the industrial revolution. Out of nine samples, only one isolate of *Pseudomonas* sp. was morphologically and biochemically identified. Presence of this isolate indicates the contamination of fresh water of lake. Coliform organisms, *Salmonella* and *S.aureus* were found absent in all the samples which indicate that quality of freshwater of the lake. However, Presence of Gram-negative psychrophylic *Pseudomonas* isolate specify that there is an urgent need to identify the source of contamination and Hygiene level of Antarctica and residing people. The research outcome also states the requirement of evaluation of Pathogenecity of the isolate and molecular identification for its further use in Science and Technology Globally.

## Additional files

10.1186/s40064-015-1354-3 Morphological and Biochemical tests carried out for further identification.
10.1186/s40064-015-1354-3 Microbiological Evaluation and Location of lake water sampling points at Stornes Peninsula.
