# Supplementary material for: Psychrophilic pseudomonas in antarctic freshwater lake at stornes peninsula, larsemann hills over east Antarctica
Source: Springerplus. 2015 Oct 7;4:582. doi: 10.1186/s40064-015-1354-3 (PMC4627980; doi:10.1186/s40064-015-1354-3)
Supplement: Supplementary file 1 — 10.1186/s40064-015-1354-3 Morphological and Biochemical tests carried out for further identification. [file 40064_2015_1354_MOESM1_ESM.docx]

**Table 1 Morphological and Biochemical tests carried out for further Identification**

| **S. No.** | **Name of test** | **Response of**  ***Pseudomonas isolate ST-2*** | **Response of**  ***Pseudomonas aeruginosa MTCC1688*** |
| --- | --- | --- | --- |
|  | Gram’s Staining | Gram Negative Rods | Gram Negative Rods |
|  | Catalase test | Positive | Positive |
|  | Oxidase test | Positive | Positive |
|  | Hugh Leifson’s test | Positive(Oxidative) | Positive(Oxidative) |
|  | Nitrate Reduction | Positive | Positive |
|  | Gelatin Liquefaction | Positive | Positive |
|  | Casein hydrolysis | positive | positive |
|  | Starch Hydrolysis | Positive | Positive |
|  | Growth at 42°C | Negative | Positive |
|  | Growth at 4°C | Positive | Negative |
