# Supplementary material for: Psychrophilic pseudomonas in antarctic freshwater lake at stornes peninsula, larsemann hills over east Antarctica
Source: Springerplus. 2015 Oct 7;4:582. doi: 10.1186/s40064-015-1354-3 (PMC4627980; doi:10.1186/s40064-015-1354-3)
Supplement: Supplementary file 2 — 10.1186/s40064-015-1354-3 Microbiological Evaluation and Location of lake water sampling points at Stornes Peninsula. [file 40064_2015_1354_MOESM2_ESM.docx]

| **Sample Location and Identification** | | | | | | **Microbiological Evaluation** | | | | |
| --- | --- | --- | --- | --- | --- | --- | --- | --- | --- | --- |
| **Sample ID** | **Date** | **Latitude (S)** | **Longitude (E)** | **Altitude (ft)** | **Temperature (ºC)** | **Psychrophilic bacterialcount/ml** | **MPN Coliform/100ml** | ***Pseudomonas* spp.** | ***Salmonella* spp.** | ***S. aureus*** |
| ST1 | 15.01.2011 | 69º 26’ 45.4” | 76º 07’ 20.6” | 155 | 1.0 | 66 cfu | NG | Absent | Absent | Absent |
| ST2 | 15.01.2011 | 69º 24’ 32.6” | 76º 07’ 36.0” | 112 | 1.8 | 1.6×10^2^ cfu | NG | Present | Absent | Absent |
| ST3 | 15.01.2011 | 69º 24’ 27.2” | 76º 07’ 15.7” | 136 | 1.6 | 55 cfu | NG | Absent | Absent | Absent |
| ST4 | 15.01.2011 | 69º 24’ 55.3” | 76º 07’ 47.6” | 108 | 1.5 | 25 cfu | NG | Absent | Absent | Absent |
| ST5 | 15.01.2011 | 69º 25’ 02.8” | 76º 07’ 26.1” | 103 | 1.2 | 22 cfu | NG | Absent | Absent | Absent |
| ST6 | 15.01.2011 | 69º 26’ 05.4” | 76º 07’ 11.4” | 122 | 1.4 | 12 cfu | NG | Absent | Absent | Absent |
| ST7 | 15.01.2011 | 69º 26’ 12.3” | 76º 07’ 19.6” | 138 | 1.1 | 22 cfu | NG | Absent | Absent | Absent |
| ST8 | 15.01.2011 | 69º 26’ 24.5” | 76º 07’ 08.9” | 148 | 1.0 | 25 cfu | NG | Absent | Absent | Absent |
| ST9 | 15.01.2011 | 69º 26’ 33.2” | 76º 07’ 31.3” | 161 | 1.1 | 23 cfu | NG | Absent | Absent | Absent |

# Table 2 Microbiological Evaluation and Location of lake water sampling points at Stornes Peninsula

Cfu: Colony forming unit, NG-No Growth Observed
